# Threefold rotational symmetry in hexagonally shaped core–shell (In,Ga)As/GaAs nanowires revealed by coherent X-ray diffraction imaging^1^

**DOI:** 10.1107/S1600576717004149

**Published:** 2017-04-13

**Authors:** Arman Davtyan, Thilo Krause, Dominik Kriegner, Ali Al-Hassan, Danial Bahrami, Seyed Mohammad Mostafavi Kashani, Ryan B. Lewis, Hanno Küpers, Abbes Tahraoui, Lutz Geelhaar, Michael Hanke, Steven John Leake, Otmar Loffeld, Ullrich Pietsch

**Affiliations:** a Faculty of Science and Engineering, University of Siegen, Siegen, 57068, Germany; b Paul-Drude-Institut für Festkörperelektronik, Hausvogteiplatz 5-7, Berlin, 10117, Germany; c Department of Condensed Matter Physics, Charles University, Ke Karlovu 5, Prague, 121 16, Czech Republic; d ESRF – The European Synchrotron Radiation Facility, 71 Avenue des Martyrs, Grenoble, 38043, France

**Keywords:** core–shell–shell nanowire, diffraction imaging, threefold rotational symmetry

## Abstract

Coherent X-ray diffraction imaging at symmetric *hhh* Bragg reflections was used to resolve the structure of GaAs/In_0.15_Ga_0.85_As/GaAs core–shell–shell nanowires grown on a silicon (111) substrate. Diffraction amplitudes in the vicinity of GaAs 111 and GaAs 333 reflections were used to reconstruct the lost phase information. It is demonstrated that the structure of the core–shell–shell nanowire can be identified by means of phase contrast. Interestingly, it is found that both scattered intensity in the (111) plane and the reconstructed scattering phase show an additional threefold symmetry superimposed with the shape function of the investigated hexagonal nanowires. In order to find the origin of this threefold symmetry, elasticity calculations were performed using the finite element method and subsequent kinematic diffraction simulations. These suggest that a non-hexagonal (In,Ga)As shell covering the hexagonal GaAs core might be responsible for the observation.

## Abstract 2

Coherent X-ray diffraction imaging was used to detect a threefold rotational symmetry in hexagonally shaped single semiconductor nanowires. The core–shell–shell structure was resolved by probing symmetric *hhh* Bragg reflections.

## 1. Introduction   

The interest in semiconductor nanowires (NWs) has been accompanied by significant improvement in fabrication and characterization techniques. New applications of NWs for electronics as well as optoelectronic and thermoelectric devices (Boukai *et al.*, 2008 ▸; Qu & Duan, 2012 ▸; Pan *et al.*, 2013 ▸; Krogstrup *et al.*, 2013 ▸) have become possible owing to their unique aspect ratio. It is obvious that the overall performance of NW-based devices is directly related to the crystal quality of individual NWs. Therefore, the control of growth parameters and careful investigation of the resulting NW quality on the level of single objects are of crucial importance. For example, the current improvement of third-generation synchrotrons together with X-ray focusing techniques has allowed researchers to correlate the electrical resistance of a single GaAs NW with the number of phase switches between zincblende and twinned zincblende phases (Bussone *et al.*, 2015 ▸). It was shown that under certain growth conditions GaAs can establish different crystallographic phases such as zincblende (ZB) with the stacking *ABCABC*… and wurtzite (WZ) with the stacking *ABAB*… (Soshnikov *et al.*, 2006 ▸; Jahn *et al.*, 2012 ▸).

One option to define core–shell heterostructures is the growth of radial NW heterostructures, which results in complicated strain distributions (Hanke *et al.*, 2007 ▸; Keplinger *et al.*, 2009 ▸; Krause *et al.*, 2016 ▸) but enables the combination of materials with different band gaps (Cingolani & Rinaldi, 1993 ▸). Pure WZ-phase GaAs/InGaAs/GaAs nanoneedles (Moewe *et al.*, 2009 ▸) and radially strained multi-shell NWs (Dimakis *et al.*, 2014 ▸) grown on Si substrates have already been demonstrated. The growth of NWs with large lattice mismatch (Caroff *et al.*, 2009 ▸) also enabled the integration of III–V semiconductors in Si technology. Widely used growth techniques for semiconductor NWs are metal–organic chemical vapour deposition and molecular beam epitaxy (MBE) (Fontcuberta *et al.*, 2015 ▸) using the vapour–liquid–solid (VLS) growth mechanism (Wagner & Ellis, 1964 ▸) where the NW diameter is defined by the radius of the liquid alloy droplets. For radial III–V NW heterostructures the growth can also be influenced by the growth on polar crystal surfaces, as was shown by high-resolution scanning electron microscopy (HR-SEM) and transmission electron microscopy (TEM) studies of GaAs NWs grown on a (111) B GaAs substrate (Zou *et al.*, 2007 ▸; Verheijen *et al.*, 2007 ▸; Paladugu *et al.*, 2008 ▸; Joyce *et al.*, 2010 ▸; Zheng *et al.*, 2013 ▸); a polarity-driven growth mechanism can favour certain facets and therefore result in locally different shell thicknesses and deviations from hexagonal shape symmetry. Despite the current progress in two-dimensional and three-dimensional imaging of the structure of nano-objects (Favre-Nicolin *et al.*, 2010 ▸; Dzhigaev *et al.*, 2016 ▸; Miao *et al.*, 2012 ▸; Pateras *et al.*, 2015 ▸; Ulvestad *et al.*, 2015 ▸), coherent X-ray diffraction imaging (CXDI) from single NWs, where strain can play a detrimental role in defining the optoelectronic properties, is still challenging for structures composed of more than one material. In particular, CXDI analysis of NW heterostructures suffers from the impact of a non-uniform strain distribution (Keplinger *et al.*, 2009 ▸; Fohtung *et al.*, 2012 ▸). The analysis of the complete strain tensor furthermore requires the measurement of several Bragg reflections (Favre-Nicolin *et al.*, 2010 ▸; Diaz *et al.*, 2010 ▸; Newton *et al.*, 2010 ▸).

In the present work we show that the core–shell–shell structure of GaAs/In_0.15_Ga_0.85_As/GaAs grown on Si(111) can be resolved by CXDI when the NW is probed in the symmetric Bragg *hhh* geometry, exploring amplitude and phase information. We recorded the CXDI patterns from different wires at GaAs 111 and GaAs 333 reflections. Considering that the radial structure is homogeneous along the NW growth axis we reduce the problem of phase retrieval (PR) to the solution of the NW structure in the (111) plane. It turns out that the majority of investigated individual NWs show trigonal plane symmetry superimposed with the hexagonal shape function of the rod itself.

The coherent scattering amplitude from a strained illuminated object in the kinematic approximation is given by 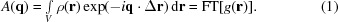
In equation (1) 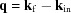 is the momentum transfer vector given by the difference between the outgoing and incoming wavevectors, and 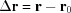 is the displacement field, where **r** is the direct-space atomic position and 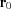 is the perfect (unstrained) lattice atomic position. The Fourier transform (FT) of the complex electron density 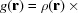 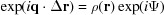 which includes the displacement field defines the scattering amplitude. In a CXDI experiment, however, only the intensity can be recorded:
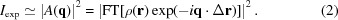
From equation (2) it is clear that the illuminated object’s shape function 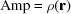 and phase Ψ in real space cannot be obtained *via* the inverse FT as the phase of the scattering amplitude is lost during measurement. Successful retrieval of the lost phase from the diffraction pattern allows access to the internal displacement field of illuminated objects with resolution of the order of a few picometres (Labat *et al.*, 2015 ▸). Thus, application of CXDI to single NWs can be used to study the detailed structure of the probed NW. By solving the two-dimensional phase problem using well known PR algorithms such as Error-Reduction (ER) (Fienup, 1982 ▸), Hybrid Input Output (HIO) (Fienup, 2013 ▸) and Shrinkwrap (SW) (Marchesini *et al.*, 2003 ▸), here we determine the displacement field of radial core–shell–shell structures and observe the appearance of trigonal shape symmetry in the phase pattern for most of the inspected NWs. Moreover, we show that the deviations in the retrieved phase from sixfold rotational symmetry around the NW growth axis are associated with a trigonal symmetry with respect to one of the 〈112〉 directions. In conventional diffraction experiments with incoherent X-rays only the average structure of the illuminated object is obtained.

## 2. Sample details and experimental geometry   

GaAs/In_0.15_Ga_0.85_As/GaAs core–shell–shell NWs were grown by MBE on Si(111) substrates covered by a ∼10 nm-thick oxide mask with lithographically defined holes. The diameter of the GaAs core and the thicknesses of the In_0.15_Ga_0.85_As and GaAs shells are 130–140, 10 and 30 nm, respectively. The GaAs NW cores were realized in two steps. First, Ga-assisted VLS growth was carried out at 903 K substrate temperature, producing GaAs NWs with 2.5 µm length, ∼50 nm diameter and mostly ZB structure. Second, the VLS Ga droplet was consumed by exposure to As_2_ and the substrate temperature reduced to 683 K, where vapour–solid GaAs growth was carried out on the NW sidewalls (Dimakis *et al.*, 2014 ▸) to increase the diameter of the NW cores. The In_0.15_Ga_0.85_As and GaAs shells were subsequently grown under similar conditions to the inner GaAs shell. As the resulting core–shell–shell structure is coherent, the (In,Ga)As shell shares a single lattice parameter with the GaAs core and outer shell along the growth axis as well as within the respective interface plane. Consequently, the (In,Ga)As is compressed biaxially by these epitaxial constraints and mostly relaxes in the radial direction.

**Figure 1:**
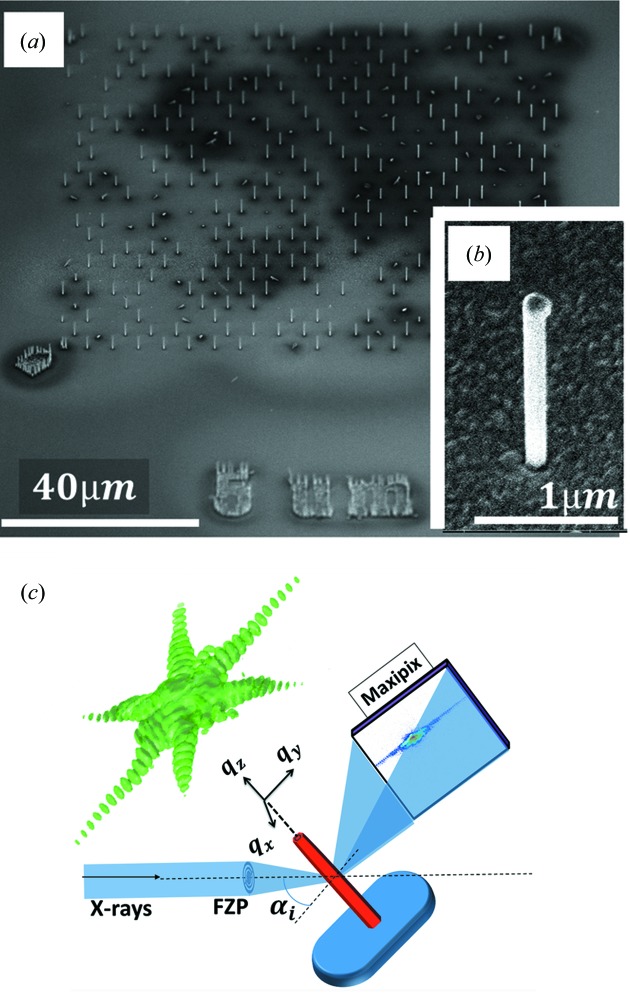
(*a*) SEM image of the array of NWs with a spacing of 5 µm. (*b*) SEM image of a single wire. (*c*) Experimental geometry for the CXDI experiment in Bragg geometry. The three-dimensional reciprocal-space map corresponds to the GaAs 111 reflection measured from NW1.

In our study an NW spacing of 5 µm was used. Several individual NWs have been investigated by means of CXDI at the ID01 beamline of the European Synchrotron Radiation Facility (ESRF), Grenoble, using a Fresnel zone plate to focus the X-ray beam with an energy of 9 keV, resulting in a 150 × 250 nm (vertical × horizontal) FWHM of the X-ray spot. By changing the angle of incidence we record a number of diffraction patterns with a two-dimensional detector (Fig. 1 ▸ *c*), thus sampling a three-dimensional reciprocal-space volume around the chosen Bragg peak. To transfer the measured data from the laboratory coordinate system to reciprocal space the approach described by Kriegner *et al.* (2013 ▸) was used. CXDI patterns near the symmetric GaAs 111 and GaAs 333 Bragg reflections for different NWs have been collected. Details of the GaAs 111 and GaAs 333 patterns are discussed in the next section. Since the footprint of the incident X-ray beam along the NW growth direction is smaller than the length of the NW, we have illuminated only a section of the NW. A scan along the growth direction verified the homogeneity of the illuminated NWs. A three-dimensional reciprocal-space map (RSM) of the GaAs 111 reflection is shown in Fig. 1 ▸(*c*). The sample geometry is such that 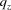 coincides with the NW growth direction, and the 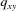 plane is defined by 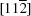 and 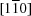 crystallographic directions.

## 3. CXDI on single wires   

**Figure 2:**
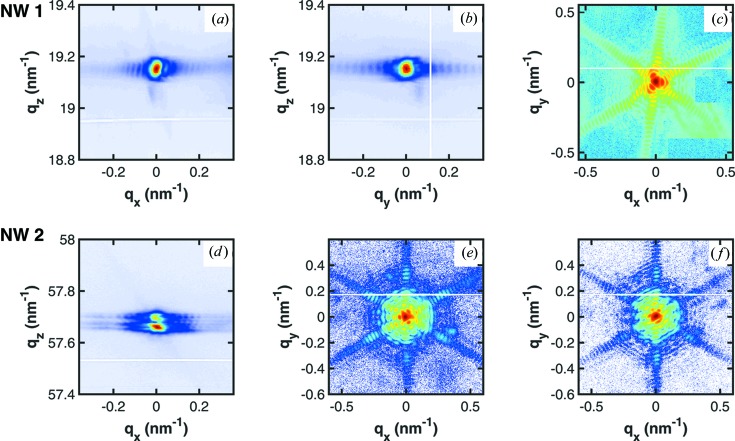
RSMs from two single wires measured at symmetric Bragg reflections. (*a*), (*b*) Projections of the three-dimensional RSM from NW1 measured at the 111 Bragg reflection. (*c*) Slice from the three-dimensional RSM in the 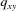 plane which is used in PR. (*d*), (*e*), (*f*) Projections of the GaAs 333 reflection for NW2. (*e*) and (*f*) show slices from the top and bottom peaks in the 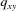 plane.

In the following we describe the results obtained by CXDI from two individual NWs. One of the NWs was measured in the vicinity of the GaAs 111 reflection; the second NW was probed at the GaAs 333 reflection where the momentum transfer is three times larger. The resulting experimental data are presented in Fig. 2 ▸, where the projection of the three-dimensional RSM onto the 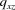 (Fig. 2 ▸ *a*) and 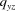 (Fig. 2 ▸ *b*) planes is shown for NW1.

The first surprising observation to be made from the diffraction patterns in the figure is the threefold symmetry of the signal in the 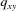 plane (Figs. 2 ▸ *c* and 2 ▸ *e*). It is most pronounced in the case of the GaAs 111 diffraction pattern shown in Fig. 2 ▸(*c*) where the central part close to 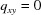 appears triangular. A further peculiar feature of the measurements is that for NW1 only a single peak is recorded along the 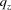 axis, whereas for NW2 a splitting of the peak is observed at the GaAs 333 reflection (see Fig. 2 ▸ *d*). There are two possible interpretations. Firstly, we have illuminated different segments of different NWs where the crystallographic phase composition is different. When using a coherent X-ray beam small fractions of WZ have a drastic influence on the peak shape. Secondly, the larger momentum transfer at the GaAs 333 reflection enhances the visibility of the peak splitting. The comparison of the 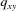 cuts produced at the maxima of the two peaks for NW2 (Figs. 2 ▸ *e* and 2 ▸ *f*) shows that the resulting RSMs are almost identical and therefore would exhibit similar direct-space features. The PR results from the GaAs 333 reflection of NW2 are therefore only shown for the RSMs in Fig. 2 ▸(*e*).

**Figure 3:**
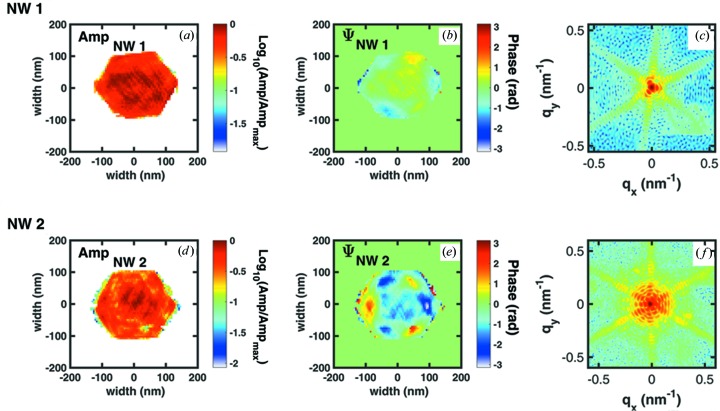
Reconstructed cross section of two different NWs from PR, where Amp stands for the amplitude of the reconstructed complex object in real space. (*a*) and (*d*) show the normalized amplitude of the NW and (*b*) and (*e*) show the reconstructed phase in two dimensions for NW1 and NW2, respectively. (*c*) and (*f*) represent the reconstructed reciprocal-space amplitude.

**Figure 4:**
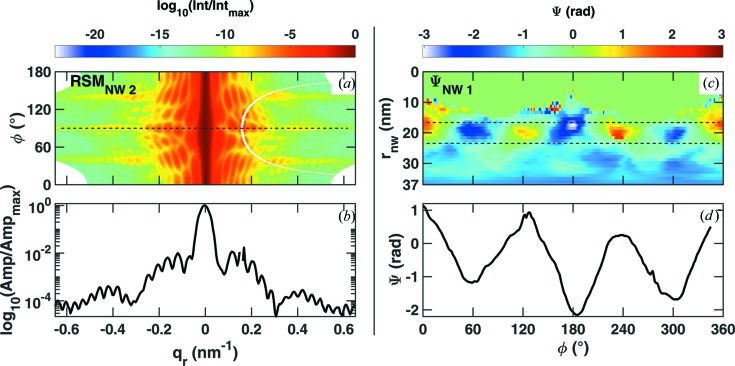
Description of the diffraction pattern and retrieved phase pattern in radial coordinates. (*a*) Representation of the diffraction pattern in Fig. 2 ▸(*f*) in radial coordinates from NW2, showing thickness oscillations (*b*) corresponding to around 30 and 220 nm thickness values which are very close to the total thickness and outer thickness of the GaAs shell. (*c*) Phase pattern from NW2 shown in Fig. 3 ▸(*d*) represented in radial coordinates, showing the appearance of different phase values at the corners of the hexagon resulting in the linear plot demonstrated in (*d*).

For PR we applied the same algorithm used by Davtyan *et al.* (2016 ▸). For the present data a combination of ER, HIO and SW iterative dual-space algorithms applied to the experimentally measured diffraction amplitudes retrieved the lost phase information from the two NWs (see Fig. 3 ▸). Given the range of 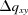 the real-space pixel size is around 5.7 nm per pixel for the retrieved electron density and phase patterns shown in Fig. 3 ▸. The retrieved amplitude shows hexagonally shaped objects with a size of 220 ± 20 nm for NW1 and 230 ± 20  nm for NW2. The phase pattern displays a predominantly threefold symmetry for the two retrievals shown in Figs. 3 ▸(*b*) and 3 ▸(*d*). The threefold symmetry of the phase patterns is correlated with the threefold symmetry of the experimental diffraction pattern shown in Fig. 2 ▸. In order to measure the structural data from the thickness fringes, we replotted the diffraction pattern shown in Fig. 2 ▸(*f*) as a function of the radial distance from the centre, 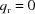, and the azimuthal angle, φ, both taken from the 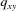 plane. The respective line scan along 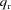 provides the total thickness of the wire (around 220 nm thick) and the outermost GaAs shell (around 30 nm thick). Considering the nominal diameter of the GaAs core to be 140 nm the thickness of the (In,Ga)As shell can be deduced to be 10 nm, in agreement with nominal values from growth. A similar procedure was applied to monitor the azimuthal phase variation, shown in Fig. 4 ▸(*c*), using the retrieved phase pattern shown in Fig. 3 ▸(*d*). The respective line cut shown in Fig. 4 ▸(*d*) represents a summation of phases taken from the region between the two black lines in Fig. 4 ▸(*c*). It shows that the phase changes from positive to negative values at each adjacent corner. Therefore, we emphasize that these phase variations are a direct result of scattering from the parts of the wire strained due to the presence of the thin (In,Ga)As shell. Because of the higher sensitivity to strain at the GaAs 333 reflection radial plots are presented for NW2. Qualitatively similar features are obtained for the NWs measured at the GaAs 111 reflection.

## 4. Finite element method modelling   

In order to find the origin of the threefold symmetry inherent to the experimentally recorded RSMs and the retrieved phase patterns shown in Fig. 3 ▸, we performed elasticity/strain calculations using the finite element method (FEM). The obtained deformation field served as input for subsequent kinematic scattering simulations. The great strength of the FEM compared with other techniques is the ease of implementing arbitrary model shapes and chemical compositions. Moreover, the FEM simulations take into account the full anisotropy of the elastic constants which map the strain to the stress tensor according to Hooke’s law. The elastic constants of In_*x*_Ga_(1−*x*)_As are retrieved *via* linear interpolation between those of GaAs and InAs following Vegard’s rule. Within the framework of linear elasticity it is thus possible to simulate the deformation field in the GaAs/(In,Ga)As/GaAs core–shell–shell NWs. However, because the FEM is a non-atomistic theory the deformation field, **u**, evaluated at the nodal coordinates of the FEM mesh cannot directly serve as input for the kinematic scattering sum: 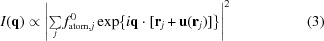
with 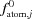 as the atomic scattering factor for the atom at the respective lattice site. To use equation (3) the deformation field has to be interpolated with an atomistic model to obtain the displacement 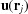 at the atomic position 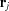 of the crystal lattice. Taking the atomic positions in the NW and their displacement as input, the scattered intensity around Bragg reflections can be computed as a function of the reciprocal-lattice vector **q**.

**Figure 5:**
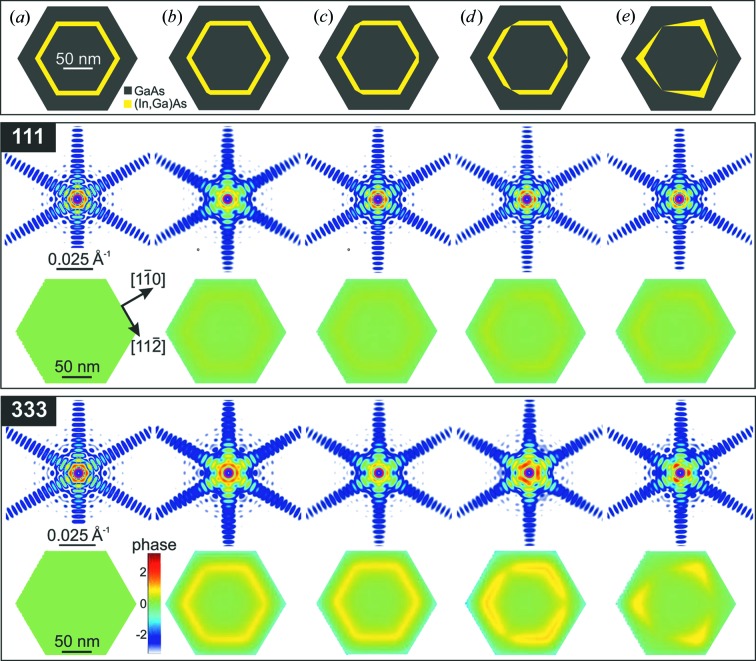
FEM models and corresponding simulated GaAs 111 and GaAs 333 RSMs and real-space phases for the single NW with different inner shell (In,Ga)As. (*a*) The (In,Ga)As shell is hexagonally shaped and the resulting RSMs and phases show sixfold symmetry. (*b*)–(*d*) The hexagonal symmetry of the (In,Ga)As shell breaks by introducing 〈112〉*A* faceting at the corners of the (In,Ga)As shell. (*e*) The deformation of the (In,Ga)As shell where almost no 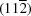 and 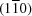 facets are visible.

For the origin of the threefold symmetry, different underlying physical scenarios have been explored. For cubic core–shell NWs with circular cross section, for example, it has been demonstrated that the shear strain components reveal sizeable trigonal symmetry when there is a lattice mismatch of 3.15% and the thicknesses of the core and shell are comparable (Ferrand & Cibert, 2014 ▸). In our case, however, the volume of the shell and the core–shell mismatch (∼1%) are considerably smaller, which makes this effect negligible. We also note that the NW shape function has sixfold symmetry, which further masks the intrinsic threefold symmetry due to the ZB structure. However, it is well known that GaAs NWs exhibit a variety of crystal phases formed within a single NW. Next to the favoured ZB phase additional polytypes are the WZ phase and twinned zincblende (Biermanns *et al.*, 2012 ▸) or even a more complicated hexagonal type of structure (Jacobsson *et al.*, 2015 ▸). This multiphase behaviour was considered in our FEM calculation by interpolating the deformation fields created by the different phases at atomic positions. This offers, at the same time, the possibility to create various models including different crystallographic phases simultaneously and to test the resulting RSMs for possible features with threefold symmetry. However, by our calculations we found that no combination of the mentioned crystallographic phases breaks the purely sixfold symmetry. Further, a variation of the shape of either the core, the (In,Ga)As shell or the GaAs shell could be thought of as a possible scenario. The core, however, is expected to be able to establish its hexagonal equilibrium shape as it only needs to facilitate the strain originating from the Si substrate. This strain is expected to be relieved after several nanometres of growth. SEM investigation of the studied NWs showed that they are of a hexagonal shape with no significant deformation, which leaves a deformation of the (In,Ga)As shell as the remaining candidate. We have found two possible scenarios for having threefold symmetry in the diffraction amplitude and real-space phase pattern for the simulated FEM models of NWs. First, we found that if every second corner shows a less expressed (In,Ga)As shell, the resulting RSM does indeed show threefold symmetry. The evolution from no deformation to a strong deviation of the (In,Ga)As shell, similar to what was observed by Zheng *et al.* (2013 ▸), is shown in the top row of Fig. 5 ▸. The middle row shows the respective simulated RSMs of the GaAs 111 reflection and the bottom row the GaAs 333 reflection. All RSMs display horizontal slices in the 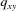 plane; the phase patterns of the simulated RSMs are shown below each respective RSM.

Column (*a*) presents the simulated data for a regular core–shell NW with no deformation of the shells. Hence, both the simulated diffraction patterns and the corresponding phase patterns only show features of the rod’s hexagonal shape function. However, the situation changes with increasing deformation of the (In,Ga)As shell. Even with the moderate local variation of the (In,Ga)As shell presented in column (*c*) the GaAs 333 reflection shows slight features of threefold symmetry, which gain in strength for models (*d*) and (*e*). In model (*e*) there is hardly any (In,Ga)As shell present in the vicinity of every second corner. This results in a clearly visible threefold symmetry in scattered intensity around the main peak. Also, the retrieved phase pattern clearly shows threefold symmetry and the phase patterns for models (*d*) and (*e*) fit very well to the ones obtained from the experimental data shown in Fig. 3 ▸.

**Figure 6:**
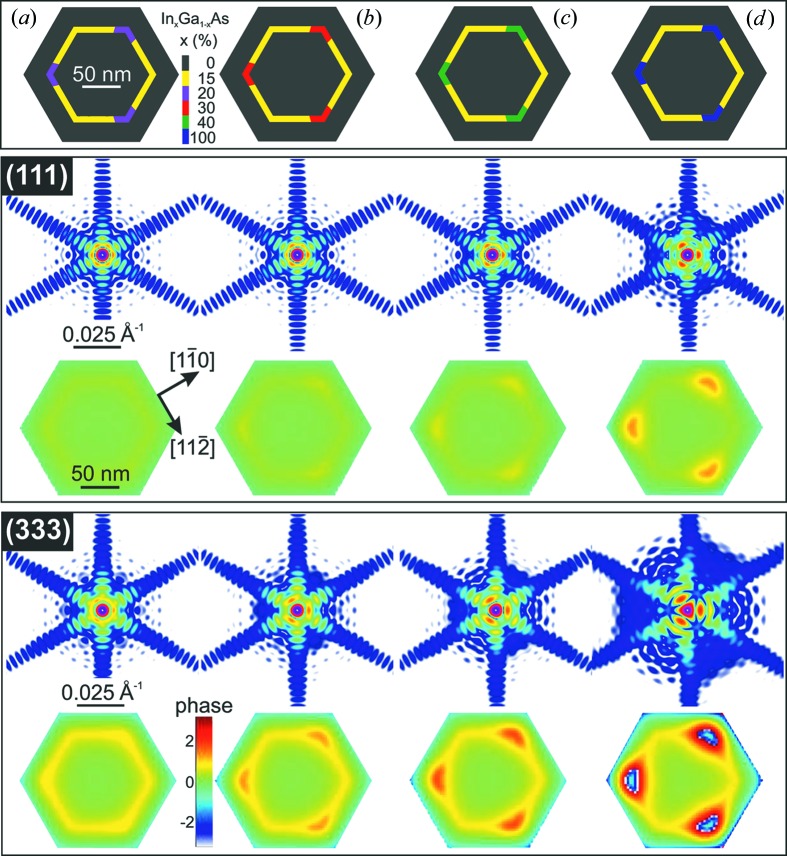
FEM models and corresponding simulated GaAs 111 and GaAs 333 RSMs and real-space phases for the single NW with a different percentage of InAs in each second corner of the inner shell (In,Ga)As. (*a*) The (In,Ga)As shell has 20% In concentration in each second corner, and the threefold symmetry in the resulting RSMs and phases is less expressed in comparison with (*b*)–(*d*), where the hexagonal symmetry of the (In,Ga)As shell breaks by increasing the In concentration from 30 to 100% at each second corner of the (In,Ga)As shell.

The second possible scenario to obtain threefold symmetry in diffraction amplitude by means of FEM modelling is given by In segregation resulting in In-rich clusters. Using the FEM it is possible to include such clusters in the model and again test the resulting RSM for threefold symmetry. We found that different concentrations of In in the clusters, *i.e.* In-rich volumes on every second corner, can introduce the threefold symmetry in the GaAs 111 or GaAs 333 reflections. For the first model we inserted In clusters with 20% In concentration on each second corner of the homogeneous 15% (In,Ga)As shell (Fig. 6 ▸ *a*). It can be seen from the RSM in Fig. 6 ▸ that the resulting symmetry is threefold, being in agreement with respective FEM phase images from GaAs 111 and GaAs 333. The concentration of In was increased further up to 30 and 40% (shown in Figs. 6 ▸ *b* and 6 ▸ *c*). The threefold symmetry of the FEM phase model becomes more expressed, even at 30% In concentration in In-rich regions. The phase changes between ±π/2 for both the FEM models and the retrieved phase information as seen in Fig. 4 ▸. Thus, the corresponding phase changes found in the corners of the hexagonally shaped NW can be correlated with the ones obtained by FEM modelling, where the latter are used for a rough estimate of the strain energy density. For the simulation shown in Fig. 5 ▸ the magnitude of the strain energy density map is of the order of 10^7^ (J m^−3^).

A possible explanation for the occurrence of such deformations can be found in the literature. It is known that GaAs grown along the [111] direction has a polar in-plane direction, namely 〈112〉, which was found to lead to non-homogeneous shell growth of core–shell NWs in similar material systems (Zou *et al.*, 2007 ▸; Zheng *et al.*, 2013 ▸; Davies *et al.*, 2015 ▸). For example, Paladugu *et al.* (2008 ▸) reported an InAs shell grown on [111]-oriented GaAs NWs and observed that InAs incorporation is higher on the 〈112〉*A* facets compared with the 〈112〉*B* facets. In our case the 〈112〉 directions point towards the corners of the wire and therefore could explain different In-shell thicknesses or concentrations in the vicinity of the corners. Therefore, polar growth can indeed be one possible scenario explaining the deviations of the (In,Ga)As shell from its hexagonal shape as presented in Fig. 5 ▸. At present we cannot judge whether the deformation originates from local variations of In content or simply local variations of shell thickness. However, NWs from two similar samples were measured by TEM and no indication of threefold symmetry was found (Grandal *et al.*, 2014 ▸). At the same time, on these samples irregular deviations from the hexagonal symmetry were common and were attributed to shadowing effects during shell growth. We stress that during the CXDI measurements of about ten NWs only two of them did not show a clear sign of trigonal symmetry, which seems to imply that shadowing effects are less relevant on the sample measured by CXDI than on the sample analysed by TEM. Further experiments will have to be performed in order to judge whether our findings are a major characteristic or restricted to a certain area of the sample under investigation.

## 5. Conclusion   

In conclusion we have shown that CXDI applied at symmetric *hhh* Bragg reflections is a powerful tool for structure identification from complex core–multi-shell NWs. From the CXDI measurement we recognize the presence of the inner GaAs and most outer GaAs shells which appear in the diffraction pattern with characteristic thickness fringes. Despite the negligible difference in electron density of the core GaAs and the inner shell (In,Ga)As, CXDI can still recognize the presence of the inner (In,Ga)As shell by means of phase contrast. The appearance of threefold rotational symmetry in diffraction patterns suggests that the (In,Ga)As shell is not homogeneous but appears with a thicker shell thickness and/or In enrichment in every second corner. Both assumptions are supported by FEM-based kinematic scattering simulations taking different degrees of deformation into account. The phase patterns for both models, shape-induced and In-rich cluster formation, from the simulated diffraction patterns show good agreement with those obtained from experimental data, suggesting a substantial (In,Ga)As shell deformation.
